# Digitizing Diagnoses: Distinguishing Infantile Hemangiomas From Other Vascular Anomalies

**DOI:** 10.1111/pde.70008

**Published:** 2025-08-14

**Authors:** Aretha On, Elena Huang, Jessica Hills, Jared Pasternak, Joanne Alfandre, Griffin Stockton Hogrogian, Albert C. Yan

**Affiliations:** ^1^ Perelman School of Medicine at the University of Pennsylvania Philadelphia Pennsylvania USA; ^2^ Primary Care Network Children's Hospital of Philadelphia Philadelphia Pennsylvania USA; ^3^ Division of General Pediatrics Children's Hospital of Philadelphia Philadelphia Pennsylvania USA; ^4^ Section of Dermatology Children's Hospital of Philadelphia Philadelphia Pennsylvania USA

**Keywords:** artificial intelligence, ChatGPT, infantile hemangioma, vascular anomaly

## Abstract

**Background/Objectives:**

Generative artificial intelligence (AI) models have become increasingly accessible and advanced with multimodal input. Infantile hemangiomas (IHs) are the most common pediatric vascular tumor, but pediatricians may have difficulty distinguishing them from similar‐appearing lesions. We assess the capability of a public AI model, ChatGPT 4.0 (GPT) to distinguish between IHs and other vascular anomalies (VAs), evaluating its potential role as a clinical tool for pediatric clinicians.

**Methods:**

This retrospective study assessed 50 IH and 50 non‐IH VA images using a GPT zero‐shot approach with the binary task of diagnosing an IH (or not). The same images were provided to four general pediatricians; comparison was performed between pediatricians and GPT.

**Results:**

GPT achieves 75% accuracy in IH identification with an *F*1 score of 0.742. ROC curve generation yields an AUC of 0.80. Hundred‐image analysis demonstrates that actual diagnosis affects GPT accuracy (*p* = 0.015). 50‐IH‐image analysis reveals configuration (*p* = 0.027), skin phototype (*p* = 0.019), and anatomical location (*p* = 0.047) as factors that may affect GPT accuracy. Comparing GPT to pediatricians reveals comparable results (*p* = 0.345).

**Conclusion:**

This off‐the‐shelf publicly available GPT (75%) was less accurate than a previously published well‐trained AI that achieved higher accuracy (~92%). GPT's *F*1 score of 0.742 indicates moderate balance between precision and sensitivity, and an AUC calculation of 0.80 indicates a potential role for GPT as a clinical assistant in primary care settings. An untuned commercial GPT does not yet outperform pediatricians despite studies of well‐trained AI. This study provides variables impacting GPT accuracy, serving as a foundation for future research.

## 1 Introduction

Infantile hemangiomas (IHs) are the most common pediatric vascular tumor, with incidence ranging from 2% to 10% [1]. They are benign, soft tissue tumors that follow a characteristic progression of development in the first few weeks of life, with proliferation and plateau over a number of months, followed by spontaneous involution [2]. Classifications of IHs include by configuration (localized, segmental, indeterminate), morphology (superficial, deep, mixed), and stage (proliferative, plateau, involution) [1, 2]. While benign and followed by spontaneous regression, IHs remain a source of parental distress and can have associated complications such as ulceration, obstruction, permanent disfiguration, and functional impairment [1, 2].

Despite being the most prevalent pediatric vascular tumor, the diagnosis of IHs can be challenging as they can resemble other vascular anomalies (VAs) [3, 4]. A study demonstrated that referral for initial diagnosis of IH had 9% of patients ultimately re‐diagnosed with a different vascular malformation, a benign anomaly, or even a malignant diagnosis (2%) (*n* = 423) [5]. Common similarly appearing lesions include various vascular malformations and vascular tumors [1, 6].

Given the need to intervene in a timely and appropriate manner, especially in cases of IH with complications, and the range in differential diagnoses, accurate diagnosis is crucial but can sometimes be difficult. Studies have investigated the role of primary care clinicians, such as pediatricians, in IH screening and the referral pathway to specialist care [7]. Various scoring tools, ultrasound, colorimetry, MRI, and biopsy have been utilized in the workup of IHs [1, 2, 7, 8, 9]. Another tool clinicians have turned to is artificial intelligence (AI) [10]. AI's emerging role in clinical diagnosis and treatment has been investigated in nearly every field of medicine [11], including in both dermatology and, more specifically, pediatric dermatology [12, 13, 14]. However, as powerful generative AI systems have become increasingly accessible to the public and as certain models have begun to incorporate multimodal input (text, photographic images, audio, and video), new studies are warranted to evaluate the appropriate role of these systems in assisting in the interpretation of clinical image data for diagnostic and therapeutic guidance. A generative AI program that launched in 2022 that shocked many people and transformed many fields, including medicine, is Chat Generator Pre‐Trained Transformer (ChatGPT) [15]. Although prior studies have demonstrated that trained AI systems can achieve high accuracy in detecting IHs [10, 16, 17, 18], the question of whether more accessible off‐the‐shelf generative AI models can perform similarly remains unanswered. We herein aim to evaluate the ability of ChatGPT 4.0 (GPT) in distinguishing between IH and other VAs via a zero‐shot approach (without prior explicit IH‐image training), to assess its potential role as a clinical tool for pediatric clinicians.

## 2 Methods

We conducted a retrospective single‐center chart review at the Children's Hospital of Philadelphia (CHOP), with IRB exemption, with all data obtained as of February 2025. All patients were < 21 years old. One hundred de‐identified photographic images of VAs were collected by pediatric dermatologists, with 50 images of IHs and 50 images of non‐IH VAs. No images were previously published or publicly available. Each image diagnosis was clinically confirmed by an experienced pediatric dermatologist (A.C.Y.). Chart reviews were performed to collect demographic information, relevant characteristics on the VA lesion (true diagnosis, anatomical location, presence/absence of ulceration, treatment at time of image) for all 100 images, and morphology, configuration, and stage for the 50 IH images.

The 100 images were randomized and presented in September 2024 to GPT using a zero‐shot approach. GPT was prompted with the binary task of determining if the diagnosis of the image was an IH or a non‐IH VA. The answers provided by GPT were recorded and analyzed to determine an error matrix and performance/reliability via accuracy, sensitivity, specificity, precision (positive predictive value), negative predictive value, and *F*1 score of GPT as a diagnostic tool. Additionally, a receiver operating characteristic (ROC) curve and area under the curve (AUC) calculation were generated from the diagnostic probabilities of IH provided by GPT for each image.

The same 100 images were randomized and presented to a cohort of four general pediatricians who were prompted with the same binary task of diagnosis of IH or not. The answers provided by each pediatrician were recorded and used to generate error matrices. A Fleiss' kappa was calculated as a measure of inter‐rater variability. Then, using a majority vote (three or more pediatricians in agreement), a pediatrician consensus dataset (PCD) was generated. The same analysis was conducted to determine the performance and reliability of the PCD, which was compared to GPT using McNemar's test.

Additionally, an analysis was conducted to evaluate the variables that potentially correlated with GPT accuracy. From the previously collected demographic information and VA characteristics, a chi‐square test of independence was performed to determine if GPT accuracy differed for given categories of the given variable. A significance level of 0.05 was used for all relevant analyses.

## 3 Results

**FIGURE 1:**
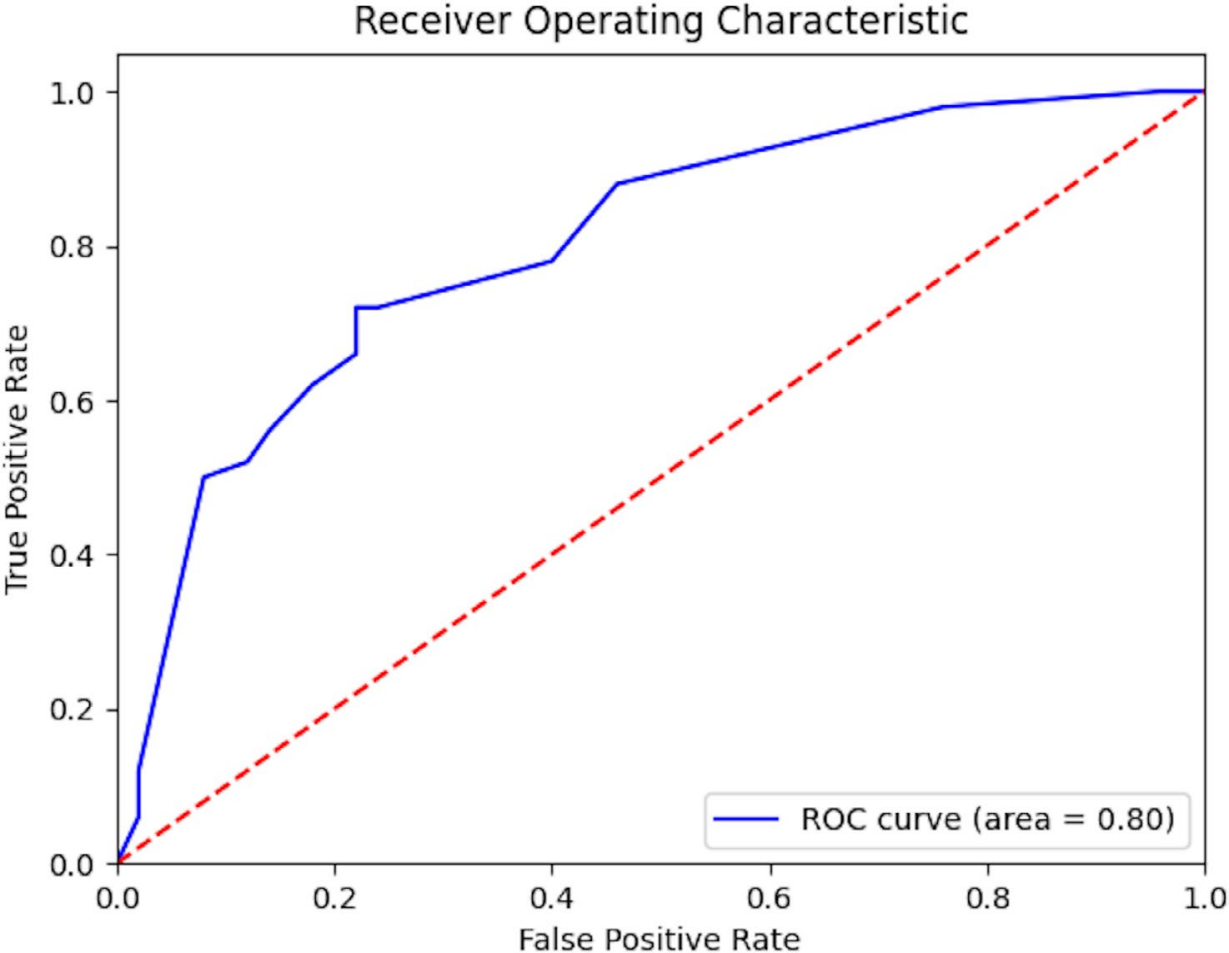
ChatGPT receiver operating characteristic (ROC) curve with area under the curve (AUC) calculation.

Demographic information of the 100 images, along with VA distribution and characteristics, is described in Table 1. Descriptive statistics determining GPT's performance and reliability are depicted in Table 2. An ROC curve generated from the probability of IH provided by GPT is depicted in Figure 1. AUC calculation was 0.80.

**TABLE 1:** Demographic information, distribution, and characteristics of vascular anomalies.

Characteristics	Value
Age at image of vascular anomaly, median (range), months	6.60 (0.07–247.13)
Sex, *n*	
Male	36
Female	61
Unknown	3
Skin phototype, *n*	
I	34
II	40
III	12
IV	7
V	6
VI	0
Unknown	1
Anatomical location, *n*	
Scalp	3
Face	44
Neck	5
Chest	11
Abdomen	4
Back	2
Groin/genitalia	2
Arm, hand	12
Leg, foot	14
Unknown	3
Diagnosis of vascular anomaly, *n*	
Infantile hemangioma	50
Congenital hemangioma, rapidly involuting	9
Congenital hemangioma, non‐involuting	1
Port wine stain	9
Venous malformation	9
Arteriovenous malformation	1
Pyogenic granuloma	9
Lymphangioma circumscriptum	4
Kaposiform hemangioendothelioma	2
Dermatofibrosarcoma protuberans	2
Tufted angioma	2
Neuroblastoma	1
Angiosarcoma	1
Unknown	0
Presence/absence of ulceration, *n*	
Presence	6
Absence	94
Unknown	0
Treatment at time of image, *n*	
Yes	37
No	46
Unknown	17
Morphology (infantile hemangiomas only), *n*	
Superficial	37
Deep	3
Mixed	10
Unknown	0
Configuration (infantile hemangiomas only), *n*	
Segmental	6
Localized	43
Unknown	1
Stage (infantile hemangiomas only), *n*	
Proliferative	33
Plateau	9
Involution	8
Unknown	0

**TABLE 2:** Performance and reliability of ChatGPT and Pediatrician Consensus Dataset (PCD).

Descriptive statistics	ChatGPT	Pediatrician Consensus Dataset (PCD)
Accuracy (%)	75	81
Sensitivity (%)	72	86
Specificity (%)	78	76
Precision (PPV) (%)	77	78
NPV (%)	74	84
*F*1 score	0.742	0.819

Abbreviations: NPV, negative predictive value; PPV, positive predictive value.

With the four general pediatricians' error matrices, Fleiss' kappa was calculated to be 0.61, which demonstrated moderate agreement between the four pediatricians. We proceeded with PCD creation with the performance and reliability depicted in Table 2. McNemar's test demonstrated no statistically significant difference between PCD and GPT performance (*p* = 0.345).

Analysis of the variables in the 100‐image full dataset determined that the VA actual diagnosis affected GPT accuracy (*p* = 0.015) (Table 3). Specifically, we found that GPT performed worse than expected (standardized residual = 3.17) on pyogenic granulomas. Analysis of the 50‐IH‐image dataset showed that configuration (*p* = 0.027), skin phototype (*p* = 0.019), and anatomical location (*p* = 0.047) were factors that affected GPT accuracy (Table 3). The standardized residuals for the IH analysis did not highlight any particular group in the variables as having a greater impact on accuracy, with the exception of GPT performing worse on skin phototype IV (standardized residual = 2.36).

**TABLE 3:** Variables correlating with ChatGPT accuracy.

Variable—full dataset (*n* = 100)	Sample size (*n*)	Chi‐square (*p* value)
Biological sex	97	0.824
Skin phototype	99	0.300
True diagnosis—IH or not	100	0.488
Actual diagnosis	100	0.015*
Anatomical location—grouped	94	0.660
Presence of ulceration	100	0.627
Treatment at time of image	83	0.845

Variable—infantile hemangioma (*n* = 50)	Sample size (*n*)	Chi‐square (*p* value)
Morphology	50	0.288
Configuration	49	0.027*
Stage	50	0.771
Skin phototype	50	0.019*
Anatomical location—grouped	49	0.047*
Presence of ulceration	50	0.889
Treatment at time of image	49	0.899

*Note*: Actual diagnosis: as described in Table 1. Configuration: segmental, localized. Skin phototype: I–V. Grouped anatomical location: face, trunk, limbs.

## 4 Discussion

The importance of early, accurate IH diagnosis results in a need to determine best practices for improving patient care, leading to some groups calling for increasing AI use in dermatology and pediatric dermatology. Studies discuss human‐AI symbiosis and AI in dermatology, especially given image recognition functions [12, 13]. Given the limited number of, yet high demand for, pediatric dermatologists, the use of AI could help address health disparities and act as a resource in underserved communities [14]. While studies have evaluated well‐trained AI in achieving high accuracy in IH detection [10], these systems are proprietary and not easily accessible to the public [16, 17, 18]. While the goal may be for these systems to be available to the public in the future, we are not at that stage. On the other hand, ChatGPT gathered over a billion monthly users just 4 months after its launch [15]. Our evaluation of GPT performance in IH diagnosis demonstrated 75% accuracy, impressive for an off‐the‐shelf model, but less accurate compared to well‐trained systems that achieved 91% [10, 17], 91.7% [10, 18], and 93.8% [10, 16] accuracy. Given the many applications of ChatGPT not only in medicine, it is logical that GPT accuracy would be lower than AI models trained to diagnose IHs [15]. However, we suspect future iterations of ChatGPT will perform better. Already, GPT 4o1, a specialized version of ChatGPT 4.0 that comes with improved coding, reasoning, and ability to follow complex instructions, has become available for text‐based input with embedded reasoning, likely improving its diagnostic capabilities, especially once combined with potential future acceptance of multimodal input [19]. Currently, GPT's *F*1 score of 0.742 indicates moderate balance between precision and sensitivity, while an AUC of 0.80 suggests reliability is good, but not adequate. This study provides novel information regarding the performance of an easily accessible, publicly available, off‐the‐shelf generative AI modality, GPT, to add to the literature.

While studies have evaluated the role of AI and ChatGPT in the clinical setting and several are in support of AI implementation [12, 13, 14, 15], limitations should be considered [15]. Concerns and criticisms of AI/ChatGPT include ethical issues, sensitivity/privacy of patient information, issues ranging from the reproducibility of ChatGPT to the phenomenon of AI hallucinations, intrinsic biases based on training data, and the generalized answers provided by ChatGPT that can lack specific indicators necessary to guide clinical practice [15, 20]. A commonly discussed phenomenon is the “black box” nature or lack of explainability of certain AI models that results in considerable ambiguity in decision‐making processes, presenting significant problems in the area of medical applications where clear explanations are vital [10]. Understanding these limitations is essential in an era where widespread accessibility of public platforms such as ChatGPT may lead new parents of children with vascular birthmarks to seek out advice from these sources [15]. Given GPT's 75% accuracy, 0.742 *F*1 score, and 0.80 AUC, caution should be exercised in the interpretation of GPT diagnosis, especially if the user is a patient/parent without a medical background. However, GPT may prove useful as a clinical assistant in the primary care setting. While GPT and PCD comparison demonstrated comparable performance, GPT notably does not yet outperform pediatricians despite studies suggesting the existence of well‐trained AI delivering high rates of accurate diagnoses [10]. General pediatricians could implement AI in their IH detection pathway to decrease misdiagnosis rates, which are reportedly around 47%–69% misdiagnosis of pediatric vascular lesions at initial evaluation [18, 21, 22]. The AUC of 0.80 indicates that GPT may misclassify some cases but performs well enough to potentially be useful in assisting in clinical decision‐making and triage, especially alongside other clinical resources in the primary care setting. Additionally, general pediatricians play a role in determining the IHs that may be clinically difficult to diagnose or manage and warrant referral to a pediatric dermatology specialist. Ultimately, in light of these data, advanced specialists such as pediatric dermatologists remain necessary to synthesize the medical information available to definitively diagnose pediatric lesions such as IHs. However, ongoing monitoring and research of these models is warranted as they continue to evolve and become more sophisticated.

Given the problematic “black box” issue highlighted earlier, there is value in investigating the processes underlying AI learning and decision‐making to improve transparency and predictability [23]. In our study, we evaluated specific variables related to VAs and IHs to determine their impact on GPT accuracy. The actual diagnosis of pyogenic granuloma had a standardized residual for incorrect of 3.17, indicating GPT was worse than expected at diagnosing these, potentially suggesting that they appear more clinically similar to IHs than other VAs. IH analysis demonstrated that configuration, anatomic site, and skin phototype affected GPT accuracy. Although the standardized residuals for the IH analysis did not highlight any particular group in the variables as having a greater impact on accuracy, the data still offer valuable insights into which variables correlated with GPT accuracy. It is possible GPT can more accurately diagnose certain IH configurations (segmental vs. localized), certain anatomic locations (face vs. trunk vs. limbs), and certain skin phototypes (I–V). Our outcomes suggest accuracy may decrease for skin of color (type IV, in particular), but given the small sample size in this study, there would potentially be a greater difference seen with larger samples, which deserves further investigation. Some of these findings for GPT are consistent with previously published data on other AI models. One well‐trained AI model performed significantly better on facial IH images [18]. The same model demonstrated equal performance on superficial versus deep IHs, which was also true for GPT performance on morphology. Our study demonstrates that certain variables influence GPT performance, with some lesion types proving more challenging for GPT to accurately recognize and diagnose than others, providing a foundation for future work to enhance transparency and better understand AI decision‐making processes.

This study is the first to investigate the accuracy of a popular, publicly available off‐the‐shelf generative AI system, GPT, in differentiating between IH and non‐IH VAs. Limitations in previous studies evaluating well‐trained algorithms include the use of non‐IH images that were clinically non‐mimickers [18], but our study utilizes non‐IH images spanning a variety of clinical IH mimickers. This study also compared GPT to general pediatrician performance and determined variables affecting GPT performance, not previously published in the literature to our knowledge. Limitations of this study include the single‐center retrospective study design, a limited data set of 100 images, use of clinical diagnosis by a pediatric dermatologist to provide ground truth, and the small sample size of general pediatricians in the comparative analysis. Additionally, image quality may be affected by who the photograph is taken by, which may affect GPT performance generalizability to pediatricians, as the images in this study were taken by pediatric dermatologists. Future studies could evaluate GPT performance in larger multicenter samples and compare it to larger cohorts of primary care clinicians with photographic images taken by general pediatricians. Future work in this area might also investigate the added value that clinical history may provide in discriminating between IHs and other VAs. Additionally, future research could explore the performance of GPT and other publicly available AI systems not only in diagnosis and detection but also in risk stratification and therapeutic management.

## Author Contributions

**Aretha On:** methodology, data collection, data analysis, writing and revising the manuscript. **Elena Huang, Jessica Hills, Jared Pasternak, Joanne Alfandre, and Griffin Stockton Hogrogian:** revising and editing the manuscript. **Albert C. Yan:** conceptualization, supervision, revising and editing the manuscript.

## Ethics Statement

All data were anonymized to ensure confidentiality, and participant privacy was maintained in accordance with relevant regulations and ethical guidelines. This study was determined to be exempt from full Institutional Review Board (IRB) review at the Children's Hospital of Philadelphia (CHOP), as it meets the criteria for minimal risk and falls within one of the exempt categories defined by federal regulations.

## Consent

The authors consent to publication.

## Conflicts of Interest

A.C.Y. consults for Johnson & Johnson, Pierre Fabre, Regeneron‐Sanofi, Verrica and is an investigator for Arcutis, Aucta, Boehringer Ingelheim, and Timber (Leo). The other authors declare no conflicts of interest.

## Data Availability

The data that support the findings of this study are available on request from the corresponding author. The data are not publicly available due to privacy or ethical restrictions.
